# Bumble bee queen pheromones are context-dependent

**DOI:** 10.1038/s41598-021-96411-7

**Published:** 2021-08-20

**Authors:** Margarita Orlova, Etya Amsalem

**Affiliations:** Department of Entomology, Center for Chemical Ecology, Center for Pollinator Research, Huck Institutes of the Life Sciences, Pennsylvania State University, University Park, PA 16802 USA

**Keywords:** Chemical ecology, Social evolution

## Abstract

Queen pheromones have long been studied as a major factor regulating reproductive division of labor in social insects. Hitherto, only a handful of queen pheromones were identified and their effects on workers have mostly been studied in isolation from the social context in which they operate. Our study examined the importance of behavioral and social context for the perception of queen semiochemicals by bumble bee workers. Our results indicate that a mature queen’s cuticular semiochemicals are capable of inhibiting worker reproduction only when accompanied by the queen’s visual presence and the offspring she produces, thus, when presented in realistic context. Queen’s chemistry, queen’s visual presence and presence of offspring all act to regulate worker reproduction, but none of these elements produces an inhibitory effect on its own. Our findings highlight the necessity to reconsider what constitutes a queen pheromone and suggest a new approach to the study of chemical ecology in social insects.

## Introduction

Pheromones have been studied for decades in insects and other organisms^1^, and throughout the course of these studies were defined as chemical stimuli evoking a stereotypical response in members of the same species. This line of thought, however, has largely overlooked the fact that an organism, in addition to the studied stimulus, will inevitably perceive, and respond to a myriad of other stimuli and influences found in its environment, that can collectively be termed context.

Context can be broadly defined as entirety of environmental conditions, biotic and abiotic, in which an individual operates, including the number and identity of conspecific individuals^2^. The importance of context was studied and well recognized in mammals, even leading to a reconsideration of the term “pheromone” to reflect that they can produce a flexible context-dependent response rather than an innate stereotypical one^2,3^. The context, however, was overlooked in other organisms, despite its potential impact on the outcome of the interaction and bear fitness consequences. A stereotypical response to a signal regardless of context can pose significant risks to the fitness of the receiver: such a response can be situationally inappropriate and thus maladaptive, or the receiver may even be exploited by the signaler through dishonest signaling (e.g.^4,5^).

The importance of context in the perception of pheromones is thus yet another facet of signal honesty: the combination of elements that comprise context, is more resistant, if not impervious, to cheating than the pheromone on its own. Signal honesty is a fundamental concept in the study of signaling and has been studied in a multitude of systems^6^. Social insects represent a test case for signal honesty since, in these organisms, the majority of females refrain from direct reproduction and put their fitness at a stake by facilitating the reproduction of a single, or a few, related females (queens), and do so, being guided in many cases by chemical signals produced by the queen, brood and nestmates^7–9^. Thus, honesty of queen pheromones is of paramount importance for workers, as a worker’s response to dishonest signaling would incur enormous losses in fitness.

The role of context in the perception of chemical signals, especially pheromones regulating reproduction, has been rarely directly tested. A number of studies suggest that social context (i.e., the presence, number, identity and behavior of individuals participating in the interaction) and chemical context (i.e., the entirety of chemical stimuli present in the environment) play a role in how individuals respond to a chemical stimulus^10–12^ and that failure to respond in a context-appropriate manner can lead to suboptimal outcomes (e.g., accepting unrelated reproductives^13^). More tellingly, the importance of context in the perception of the queen pheromones is suggested by a number of studies where naturally or artificially derived components of an alleged queen pheromone produce an effect on workers but not to the extent that a live queen in a natural setting does^14–16^. Even in the seminal article stating the effects of the honey bee queen mandibular pheromone (QMP)^17^, QMP affected worker reproduction to a lesser extent than the complete colony environment with a live queen.

In this study we examined the role of context in the perception of queen chemical signals using *Bombus impatiens*. Bumble bees are excellent models to study this question since worker reproduction are sought to be regulated by a combination of behavioral and chemical means^9,18,19^, serving as an excellent test case for the effect of chemical signalling perceived in and out of behavioral and social context. Our previous study demonstrated that bumble bee queens use different strategies to regulate worker reproduction during their life cycle, with young queens relaying on aggressive behavior, while older queens may rely more heavily on chemical signals^20^. We further showed that the queen’s cuticular profile changes with her life stage. Older queens produce larger amounts of short hydrocarbons (below or equal to 24 carbons chain length) as compared to young queens, and the ratio between short and long hydrocarbons (above or equal 26 carbons chain length) predicts worker ovarian activation^20^. Here, we examined the role of these chemical extracts in regulating worker reproduction. We tested if chemical signals produced by virgin, young, and old queens, are able to regulate reproduction in and out of context of a live queen’s physical and visual presence and the presence of eggs.

## Methods

### Experimental setup

Workers were exposed to cuticular extracts of virgin, young, and old queens in three settings that included *no context* (n = 38 cages), *partial context* using a live free-moving newly-emerged virgin queen (n = 37 cages), or *full context* using a live free-moving newly-emerged virgin queen and eggs (n = 47 cages). To control for the effect of eggs alone, workers were also kept with eggs without the queen or queen extracts (n = 13 cages). Hexane controls were included in three previous studies that we conducted with *B. impatiens* queens^14,21,22^ and did not differ from the extract treatment or the lack of treatment. Thus, the current experiments were designed in a comparative way to further disentangle the influence of context, assuming the solvent had no effect by its own. Bees were kept in plastic cages (11 cm diameter × 7 cm height) that were lined up with a filter paper and contained 60% sugar solution and fresh pollen (provided every other day). All cages contained a pair of newly emerged workers with the extract and the social context element/s. Egg cells were placed directly on the filter paper. Each egg cell contains several eggs covered in wax, and all eggs remained alive throughout the experiment, as evident by the presence of live eggs or larvae at the end of the experiment (see below). All pairs were kept for 7 days before they were terminated. Extracts containing 1 queen equivalent (1 Qeq) in 5 µl of hexane were applied daily to a glass coverslip (1 × 1 cm) in the context-free treatment or to the thorax of the live virgin queen in the partial context and full context treatments. The application was repeated for 7 days. In the full context setting and in cages containing only eggs, two batches of newly laid eggs were added to the cage on days 1, 3, 5 and 7, resembling the rate of egg laying by an active queen. At the end of the experiment all cages were frozen, workers were dissected to measure ovarian activation and the presence of eggs and larvae in each cage was recorded. Cages in which the queen or one of the workers died prematurely were removed from the analysis. Cages in which no egg batches were placed (context-free and partial-context treatments) were inspected for egg laying at the end of the experiment. As intended, no eggs were laid by either the virgin queens or the workers within the duration of the experiment as workers typically lay eggs within 8–9 days^14^ and virgin queens have inactivated ovaries. The sample size and the setup are summarized in Table 1.

**Table 1:** The experimental design and sample size used in the study.

Queen extract/setting	Context-free (glass coverslip)	Partial context (live virgin queen)	Full context (live virgin queen and eggs)	Egg presence (eggs only)
Virgin queen extract	13	13	16	0
Young queen extract	12	12	15	0
Old queen extract	13	12	16	0
No extract	0	0	0	13
Total	38	37	47	13

Worker reproduction was examined in three different contexts with three types of queen extracts. The numbers within the table denote numbers of cages. Each cage contained two newly-emerged workers that were frozen on day 7.

### Bees and housing

Twelve colonies of *B. impatiens* were obtained from Koppert Biological Systems (Howell Michigan, USA) or reared in the lab using gynes from Koppert colonies as founders. Colonies maintained in the laboratory under constant darkness, a temperature of 28–30 °C, 60% relative humidity, and supplied ad libitum with a 60% sugar solution and fresh pollen (Koppert Biological Systems). These colonies were used as a source of newly emerged workers (younger than 24 h), eggs and virgin queens.

### Queen extracts

Cuticular extracts were collected from three types of queens (virgin, young and old) using a non-lethal method (see below). Newly-emerged virgin queens (n = 40) were collected from Koppert colonies and placed in separate cages upon emergence. Cuticular extracts from these queens were sampled every other day for the duration of 14 days since emergence. A portion of the newly-emerged virgin queens were aged for 6 days, mated in the laboratory with unrelated males and underwent CO_2_ treatment according to the protocol described in^23^. They were housed in individual cages until they laid eggs and produced workers. Extracts from these young egg-laying queens (n = 20) were sampled every other day for the duration of 14 days since the emergence of their first daughter. Old egg-laying queens (n = 16) were obtained from lab-reared colonies several months following the emergence of the first worker. These colonies contained > 100 workers and were producing gynes and males. Extracts were sampled from these queens every other day for the duration of 30 days since the first gyne/male emergence.

### Non-lethal sampling of queen cuticular extracts

Non-lethal sampling was performed as described in^20^. Briefly, individual queens were placed in 20 ml glass vial for 10 min. Afterward, the vial was washed with 1 ml hexane. The amount of queen’s cuticular hydrocarbons obtained in this way from one queen was termed one queen equivalent (Qeq). The extracts from ten queens of the same type were pooled, evaporated and transferred to 2 ml glass vials fitted with a 200 μl glass insert. The pools were evaporated and reconstituted with 50 μl hexane, so that 5 μl solution contained one queen equivalent. Extracts were stored at − 20 °C to prevent evaporation.

### Chemical analysis of extracts

Cuticular extracts of virgin, young, and old queens were chemically analyzed in a previous publication^24^. To confirm that the extracts are similar to previous data, we analyzed 13 pools of queens (same pools as we used in the experiments, 4–5 pools per queen type). Pools were also used to calculate the queen equivalent amount in each of these queen types. To this end, 10 µl (2 Qeq) from each pool were mixed with 50 µl hexane containing 100 ng pentadecane (Sigma) as an internal standard. The mixture was evaporated to the volume of 10 µl out of which 1 µl containing 10 ng pentadecane and 0.2 Qeq of queen secretion were analyzed using GC/MS. In addition, we collected and analyzed 36 individual samples of virgin, young and old queens (8–19 extracts per queen type) and compared the individual queen equivalent to the one calculated from the pool. The total amount of compounds was summed and averaged for each queen type.

Sample quantification was performed using gas chromatograph Trace 1310 (ThermoScientific) equipped with a TG-5 ms column. The run was performed in splitless mode with temperature program from 60 °C at 15 °C/min for 4 min to 120 °C, then at 4 °C/min for 54 min to 300 °C with a final hold of 5 min. All chromatograms were integrated using Chromeleon 7.0 software (ThermoScientific). Compounds were identified based on our previous work^24^ and by matching retention times and spectra with authentic standards. Peak areas were normalized to the internal standards.

### Measurement of ovarian activation

All workers were 7 days old upon freezing. Workers were dissected under a stereomicroscope. Ovaries were obtained and placed into drops of distilled water. The length of the terminal oocyte in the three largest ovarioles was measured with a micrometer eyepiece embedded into the lens. Workers possess four ovarioles per ovary and at least one oocyte per ovary was measured. Mean terminal oocyte length for each bee was used as an index of ovarian activation^25^.

### Statistical analysis

Statistical analyses were performed using SPSS v.21. Normal distribution of terminal oocyte size was confirmed using Kolmogorov–Smirnov test (p = 0.2). Generalized Linear Models analysis (hence GLM) was employed for comparisons of worker oocyte size. In all analyses we tested for the effect of setting (context-free, partial context and full context), treatment (extract type of virgin, young, and old queens) and the interaction between them followed by post-hoc pairwise contrast estimation using Least Significant Difference (LSD) method.

## Results

**Figure 1:**
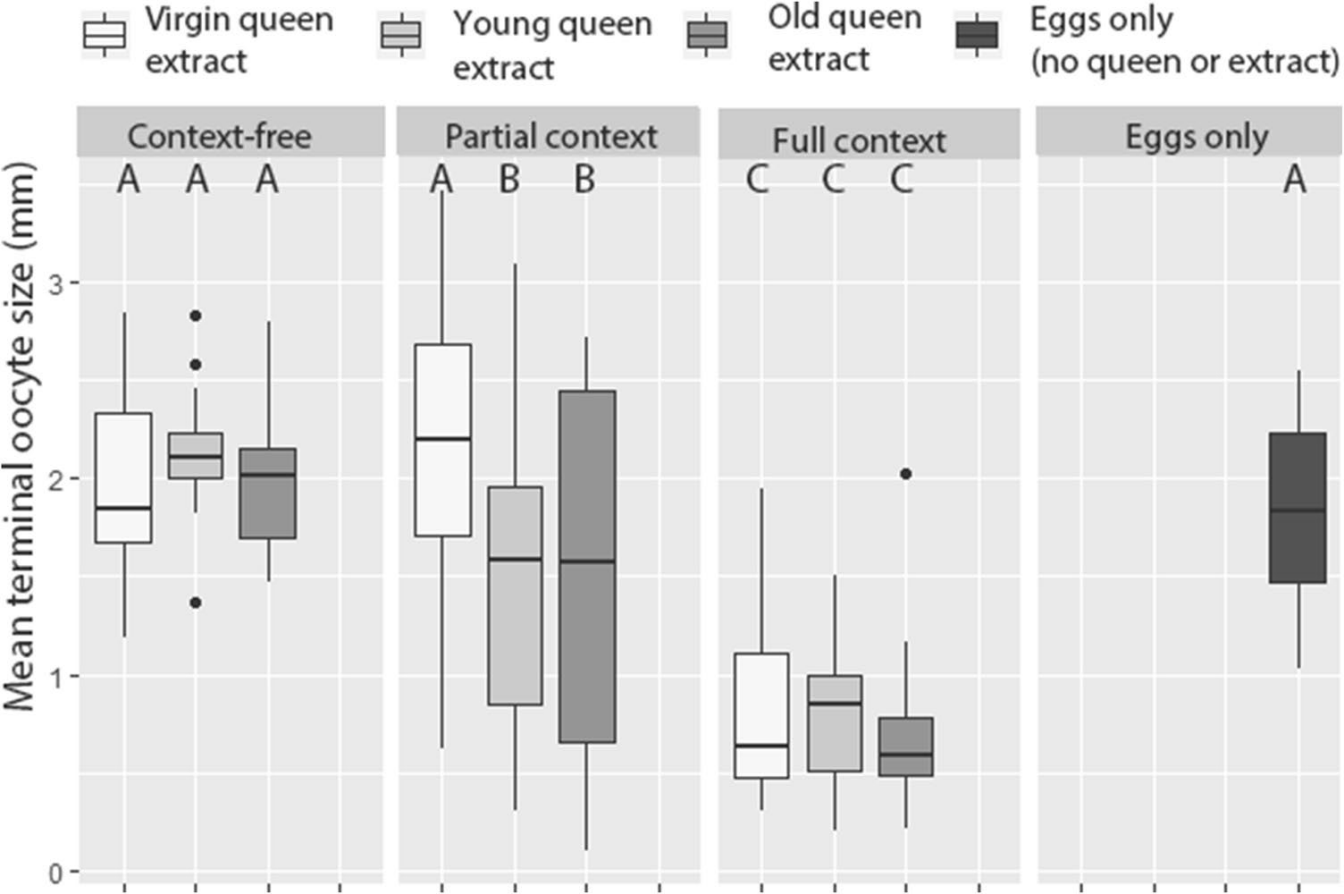
Worker reproduction in response to queen semiochemicals in and out of social context. Worker were exposed to chemical extracts of virgin, young and old queens in three different settings: where no context is provided (i.e., extracts were applied to a glass coverslip), where partial context is provided (i.e., extracts were applied to the thorax of newly-emerged virgin queens) and where full context was provided (i.e., extracts were applied to virgin queen and newly laid eggs were added to the cage every other day). The effect of exposure to eggs only without a queen or queen’s extracts is shown in a separate column. One queen equivalent of extract was provided daily for 7 days, by the end of which worker oocytes were measured. All cages contained a pair of newly-emerged workers at the onset of the experiment.

Generalized Linear Mixed Models analysis revealed a significant effect of the setting, but not of the treatment on mean worker oocyte size, however, the interaction between setting and treatment was significant (GLM, Wald χ^2^_2_ = 116.44, p < 0.001 for treatment, Wald χ^2^_2_ = 4.54, p > 0.05 for extract type and Wald χ^2^_4_ = 11.67, p = 0.02 for interaction) (Fig. 1). Post-hoc contrasts of setting revealed that workers exposed to full context (live queens and eggs) had the smallest oocyte size than any setting group (p < 0.001 for all comparisons), and in workers exposed to partial context (live queens without eggs), the oocyte size was smaller than in those exposed to context-free setting (p = 0.001). Oocyte size in workers kept in cages with eggs but without a queen or extracts did not differ significantly from that in the context-free or partial context settings (p > 0.05). Post-hoc analysis of interaction between setting and treatment revealed that only in the partial context setting, the extract type had an effect on worker oocyte size with workers exposed to extracts of virgin queens having larger oocytes than those exposed to extracts of young and old queens (p = 0.001 and p = 0.004 respectively). In the context-free and full context settings, extract type had no effect on worker oocyte size (Fig. 1).

Analysis of CHC composition in pools and individual samples from different types of queen show that peak identity is similar to our previous study^24^. It also revealed that, in accordance with our previous findings, the ratio of short to long hydrocarbons was highest in old queen extracts (ratio = 2.25) and lowest in virgin queen extracts (ratio = 0.68), while in young queen extracts the ratio was intermediate (ratio = 1.14). The list of compounds in the queen cuticular secretions and their absolute and relative amounts in the different extracts are provided in Table 2.

**Table 2:** Absolute (ng) and relative amounts (%) of compounds in cuticular extracts of queens.

	Amount (ng)			Relative amount (%)		
	Virgin	Young	Old	Virgin	Young	Old
Palmitic acid	364.12 ± 183.43	453.99 ± 144.53	419.46 ± 298.83	7.42 ± 1.62	3.54 ± 0.96	2.94 ± 1.18
Heneicosane	54.88 ± 14.57	68 ± 14.24	93.63 ± 52.48	1.16 ± 0.19	0.7 ± 0.07	0.93 ± 0.19
Oleic acid	444.94 ± 225.32	784.44 ± 217.62	1032.2 ± 763.01	12.46 ± 3.37	6.97 ± 1.22	4.13 ± 1.35
Tricosene	1047.77 ± 486.57	576.93 ± 168.47	1064.85 ± 639.05	3.21 ± 0.55	4.9 ± 0.69	12.46 ± 2.88
Tricosane	1208.25 ± 327.59	1299.32 ± 270.35	2013.37 ± 1104.57	8.08 ± 0.71	13.24 ± 0.63	19.94 ± 1.55
Tetracosene	77.01 ± 31.53	146.04 ± 39.67	166.05 ± 120.23	1.97 ± 0.38	1.37 ± 0.18	1.13 ± 0.2
Tetracosane	60.01 ± 18.48	60.44 ± 13.93	105.93 ± 64.27	0.79 ± 0.26	0.6 ± 0.04	0.83 ± 0.07
Pentacosene	1070.24 ± 313.59	1485.6 ± 381.32	1924.58 ± 1184	13.6 ± 1.05	13.13 ± 1.07	17.8 ± 1.63
Pentacosane	1153.67 ± 351.88	1223.45 ± 242.3	2036.65 ± 1238.86	12.87 ± 1.13	12.83 ± 0.76	16.82 ± 1.38
Hexacosene	13.67 ± 3.27	61.56 ± 15.15	13.21 ± 3.55	0.87 ± 0.11	0.62 ± 0.08	0.42 ± 0.1
Hexacosane	12.37 ± 3.37	48.27 ± 10.36	11.27 ± 3.49	0.7 ± 0.15	0.53 ± 0.06	0.26 ± 0.03
Heptacosene	165.5 ± 40.19	551.82 ± 121.6	272.92 ± 152.57	8.65 ± 0.78	5.67 ± 0.39	3.64 ± 0.62
Heptacosane	292.16 ± 83.59	733.18 ± 160.19	461.73 ± 260.9	10.86 ± 1.26	8.09 ± 0.8	4.62 ± 0.44
Tetracosyl acetate	228.14 ± 79.26	732.96 ± 180.71	286.77 ± 146.05	0.66 ± 0.22	7.88 ± 0.89	2.63 ± 0.74
Nonacosene	106.47 ± 26.14	494.88 ± 115.73	177.98 ± 99.58	6.27 ± 0.63	5.32 ± 0.53	2.45 ± 0.45
Nonacosane	171.45 ± 48.63	313.96 ± 65.4	286.91 ± 160.53	4.21 ± 0.3	3.06 ± 0.2	3 ± 0.38
Hexacosyl acetate	169.41 ± 50.71	505.02 ± 129.81	271.62 ± 157.78	1.28 ± 0.27	5.41 ± 0.61	1.98 ± 0.36
Hentriacontene	114.44 ± 25.26	413.93 ± 84.05	176.93 ± 87.66	3.18 ± 0.35	4.65 ± 0.39	2.77 ± 0.58
Hentriacontane	57.58 ± 20.04	86.01 ± 21.34	101.57 ± 64.12	0.72 ± 0.16	0.74 ± 0.11	1.01 ± 0.24
Octacosyl acetate	13.69 ± 4	60.01 ± 19.27	11.91 ± 3.92	1.03 ± 0.28	0.74 ± 0.12	0.21 ± 0.07

Data were collected from 13 pools (4 pools of virgin, 5 pools of young, and 4 pools of old queens, each pool contained 10 queens) and 36 individual samples (9 virgin, 19 young, and 8 old queens). Data are presented as mean ± SE.

**Figure 2:**
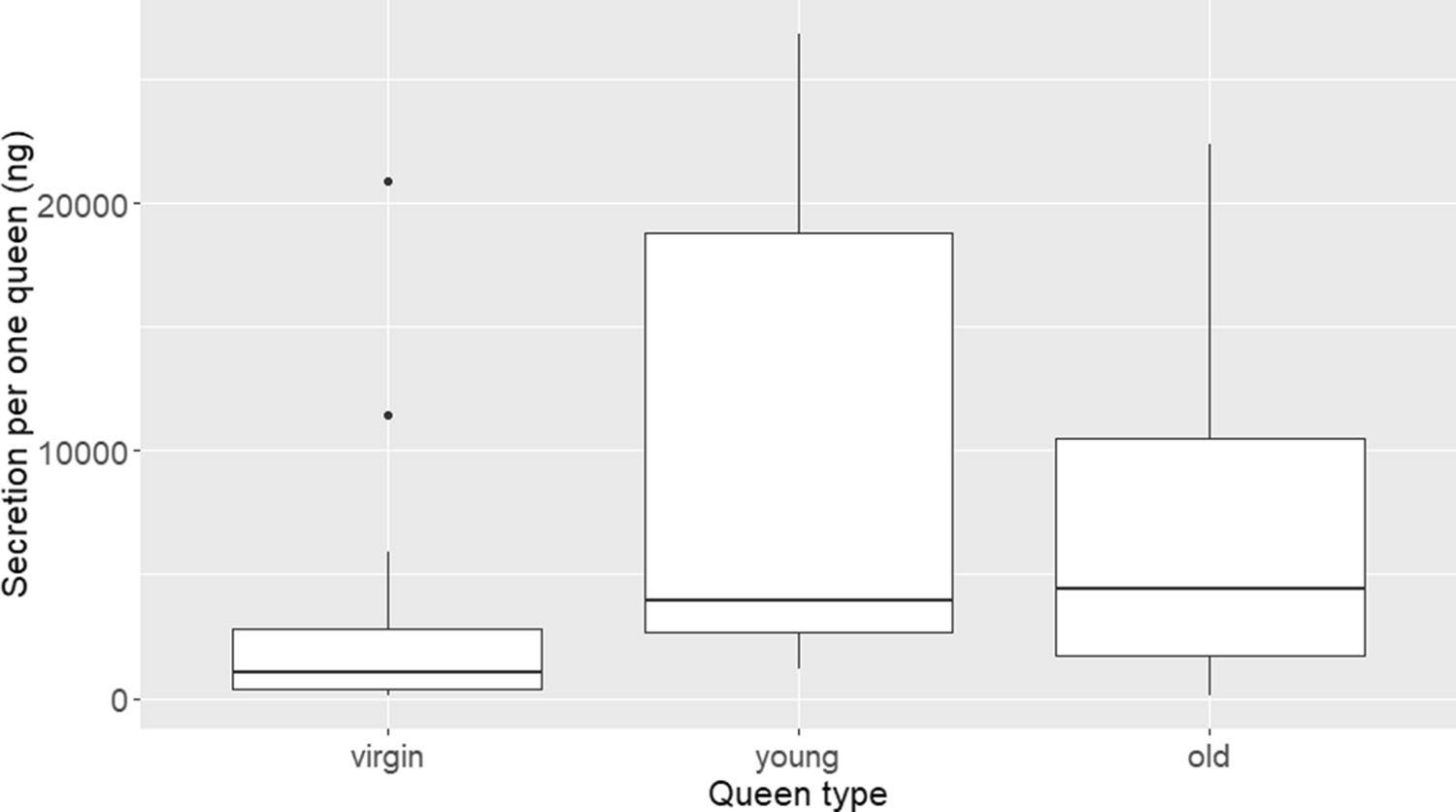
One queen equivalent amount in different queen types. The total secretion in one queen equivalent of virgin, young and old queen extracts. Data were collected from 13 pools (4 pools of virgin, 5 pools of young, and 4 pools of old queens, each pool contained 10 queens) and 36 individual samples (9 virgin, 19 young, and 8 old queens).

We further calculated the queen equivalent amount in the pools (n = 13, 4–5 per queen type) and in the individual samples (n = 36, 8–19 per queen type). The calculated one queen equivalent in both pools and individual samples was similar and the data were combined. The total cuticular lipid amounts differed significantly between queen types with young queens having, on average, the highest amounts of secretion (10.1 ± 1.9 μg), and virgin queens the lowest (3.6 ± 1.6 μg) (GEE, Wald chi-square = 6.62, df = 2, p = 0.03). Post-hoc LSD analysis revealed that young queens had significantly higher amounts of CHCs than virgin queens and CHC amounts in old queens were intermediate (Post-hoc LSD, p < 0.01 for young vs. virgin, p > 0.05 for other comparisons) (Fig. 2).

## Discussion

In this study we examined the importance of context in the perception of pheromones. Our results overall demonstrate that queen chemistry affect worker reproduction only when set in the context of the queen’s visual presence and the presence of queen’s offspring. Differences in the chemical output of queens that we identified in our previous work^24^ produce differential effects on worker physiology only when set against the background of the queen’s physical and visual presence and only between virgin to young and old queens, but not between young and old queens. Such context-dependent response to queen semiochemicals has not been previously reported in social insects but an effect of social context on the production and the preception of pheromones has been documented in Drosophila^26,27^ as well as in mammalian models^28–31^.

As expected, when applied to virgin queens, cuticular extracts of virgin queens failed to reduce worker reproduction while those of young and old queens reduced worker oocyte size. Contrary to our expectations, extracts of young and old queens applied to live virgin queens produced a similar effect on worker reproduction, and both were significantly different from those of virgin queens. This may suggest that the CHC ratio is not the only parameter reflecting the queen’s age and physiological state. Another possible explanation might be that the virgin queens that we treated as a blank slate for application of exogenous compounds are not, in fact, a blank slate. We used virgin queens for the duration of 7 days following eclosion. During this time, they may have developed their own cuticular chemistry that eventually mixed with the extracts applied to them. Virgin queens possess an abundance of long (equal or longer than 26 carbons) CHCs, and the ratio of short to long hydrocarbons on virgin queen cuticle is on average low (0.68 in the current study). Applying an extract with high short to long CHC ratio to a background where this ratio is very low will result in lowering the overall ratio of the mixture. This can, in turn, level the differences between old and young queen extracts. This possibility raises the question as to how prominent or subtle the differences in chemical profile need to be for workers to perceive them, and this question warrants further study. The differences in the total amount of queen CHC extracts (Fig. 2) may further support this explanation as young queens had on average × 3 secretion compared to virgin queens and × 2 secretion compared to old queens. If in addition to the ratio of short to long hydrocarbons, workers are also sensitive to the sheer amounts of short hydrocarbons, they may be more responsive to extracts of young queens due its overall larger quantity of CHC (Fig. 2). One way or another, it is important to note that the sheer quantity of CHCs alone cannot explain the differences between the social settings for two reasons: the first, being the lack of statistical differences between the effect of virgin queen extracts and virgin queens smeared with virgin queen extracts despite the later contains a double amount of CHCs (Fig. 1). The second reason being the lack of differences between young and old queens in their effect on worker reproduction despite a double amount of CHCs in the young compared to the old queens (Fig. 2).

Another important finding of this study is that different elements of context, such as the presence of eggs and the visual presence of a live queen, act together to affect worker reproduction to a greater extent than either a live (virgin) queen or eggs alone. This is in line with previous findings concerning the effects of queen and brood on different aspects of worker reproduction^32,33^. Our results in this case mirror the findings from another bumble bee species, where elimination of the queen’s eggs impaired her ability to regulate worker reproduction and behavior, even though the queen herself remained intact^34^. The combined effect of the queen chemistry and egg presence is especially important given that the presence of eggs is the ultimate testimony to the honesty of the queen signal. Eggs demonstrate that the queen is active and fertile. Interestingly, in the presence of eggs, all queen extracts produce the same effect, suggesting that the effect of eggs is powerful enough to override differences in queen secretions. To understand how this happens, we need better knowledge of egg chemistry and a better mechanistic understanding of how queen chemistry and egg chemistry are perceived by workers.

Overall, our findings challenge the idea of queen semiochemicals as substances acting on worker reproduction regardless of the circumstances. In most studies hitherto the main criterion by which a substance was identified as a queen pheromone was its ability to affect worker reproduction in isolation (e.g.^17,35^). This approach however overlooks the biological reality in which queen pheromones operate and dismisses a variety of compounds and blends that probably act as signals but require a contextual setting for their full effect on worker reproduction. It also ignores the weight of the decision workers need to make when tuning to the queen signals, which is to forgo reproduction and rear someone’s else offspring. Gathering information from multiple sources provides workers an insurance against cheating or exploiting their ability to perceive and respond to certain semiochmicals. The dearth of queen, brood and nestmate semiochemicals regulating reproduction that have been positively identified thus far^9^ is likely a consequence of this narrow definition. Our findings highlight the necessity for a broader view of what constitutes a queen pheromone.
